# Molecular and serological evidence of Crimean-Congo hemorrhagic fever orthonairovirus prevalence in livestock and ticks in Cameroon

**DOI:** 10.3389/fcimb.2023.1132495

**Published:** 2023-03-28

**Authors:** Huguette Simo Tchetgna, Francine S. Yousseu, François-Loïc Cosset, Natalia Bezerra de Freitas, Basile Kamgang, Philip J. McCall, Roland Ndip Ndip, Vincent Legros, Charles S. Wondji

**Affiliations:** ^1^ Microbiology and Parasitology Department, Centre for Research in Infectious Diseases, Yaoundé, Cameroon; ^2^ Department of Microbiology and Parasitology, Faculty of Science, University of Buea, Buea, Cameroon; ^3^ Centre International de Recherche en Infectiologie (CIRI), Team Enveloped Viruses, Vectors and Immunotherapy (EVIR), Univ Lyon, Université Claude Bernard Lyon 1, Inserm, U1111, Centre National de la Recherche Scientifique (CNRS), UMR5308, Ecole Normal Superieur (ENS) Lyon, Lyon, France; ^4^ Vector Biology Department, Liverpool School of Tropical Medicine, Liverpool, United Kingdom; ^5^ Campus vétérinaire de Lyon, VetAgro Sup, Université de Lyon, Lyon, France

**Keywords:** zoonosis, emerging and re-emerging virus, Central Africa, CCHFV, Dugbe virus, Orthonairovirus

## Abstract

**Introduction:**

Despite a high fatality rate in humans, little is known about the occurrence of Crimean-Congo hemorrhagic fever virus (CCHFV) in Cameroon. Hence, this pioneer study was started with the aim of determining the prevalence of CCHFV in domestic ruminants and its potential vector ticks in Cameroon.

**Methods:**

A cross-sectional study was carried out in two livestock markets of Yaoundé to collect blood and ticks from cattle, sheep, and goats. CCHFV-specific antibodies were detected in the plasma using a commercial ELISA assay and confirmed using a modified seroneutralization test. Ticks were screened for the presence of orthonairoviruses by amplification of a fragment of the L segment using RT-PCR. Phylogeny was used to infer the genetic evolution of the virus.

**Results:**

Overall, 756 plasma samples were collected from 441 cattle, 168 goats, and 147 sheep. The seroprevalence of CCHFV was 61.77% for all animals, with the highest rate found in cattle (433/441, 98.18%) followed by sheep (23/147, 15.65%), and goats (11/168, 6.55%), (*p*-value < 0.0001). The highest seroprevalence rate was found in cattle from the Far North region (100%). Overall, 1500 ticks of the *Rhipicephalus* (773/1500, 51.53%), *Amblyomma* (341/1500, 22.73%), and *Hyalomma* (386/1500, 25.73%) genera were screened. CCHFV was identified in one *Hyalomma truncatum* pool collected from cattle. Phylogenetic analysis of the L segment classified this CCHFV strain within the African genotype III.

**Conclusion:**

These seroprevalence results call for additional epidemiological studies on CCHFV, especially among at-risk human and animal populations in high-risk areas of the country.

## Introduction

Crimean-Congo hemorrhagic fever (CCHF) is endemic in Africa, eastern Europe the Middle East and in Asia and is the most widespread tick-borne disease in the world (Fanelli and Buonavoglia, 2021). CCHF is caused by an enveloped, segmented, negative sense, single-strand RNA virus (CCHFV) belonging to the *Orthonairovirus* genus, *Nairoviridae* family (Garrison et al., 2020). The viral genome consists of three RNA segments: small (S), medium (M), and large (L), which encode the viral nucleoprotein (NP), the glycoprotein precursor (GPC) which is matured in two structural glycoproteins (Gn and Gc), and the RNA-dependent RNA polymerase, respectively (Garrison et al., 2020). CCHFV is among the most genetically diverse arboviruses known currently. Many genotypes are distinguished based on the genomic segment considered and they show geographic segregation according to the origin of the virus. The phylogenetic analysis of the S segment described seven lineages named Africa 1, Africa 2, Africa 3, Asia 1, Asia 2, Europe 1, and Europe 2 while nine and six additional genetic lineages can be characterized for the M and L segments respectively, with however a certain congruent level (Deyde et al., 2006; Bente et al., 2013; Guo et al., 2017). Interestingly, segment reassortment are common with CCHFV, leading to differences in the phylogenetic three topology for the same isolate when the three genomic segments are analysed (Deyde et al., 2006; Chinikar et al., 2015).

CCHFV is a zoonotic virus maintained in an enzootic transmission cycle involving Ixodidae ticks and several vertebrate animals such as birds, small mammals, domestic animals, and wild ungulates (Kuehnert et al., 2021). Although the virus has been found in different species of ticks such as *Amblyomma* spp., and *Rhipicephalus* spp., *Hyalomma* spp. ticks are considered as the primary vectors and reservoirs of CCHFV. In fact, the area of endemicity of CCHFV closely mirrors that of the different species of *Hyalomma* ticks in Africa, Europe, and Asia with the involvement of local species (Turell, 2007; Okely et al., 2020). *Hyalomma* maintains the virus through transovarial, transstadial, and venereal transmission (Turell, 2007). CCHF infection in non-human vertebrates is usually asymptomatic or mild with a viremia lasting less than 14 days (Spengler et al., 2016). CCHFV can be transmitted to humans through the bite or crushing of an infected tick, by direct contact with blood or tissues of a CCHFV-infected animal or patient. Nosocomial transmission has been documented as mostly associated with unsuitable sterilization of medical equipment and contamination of medical supplies (Whitehouse, 2004; Turabi Gunes et al., 2009). Clinical presentation of CCHF in humans is variable, from a mild non-specific febrile illness to a fatal haemorrhagic fever characterized by disseminated intravascular coagulation, shock, and multiple organ failure. No commercial vaccines and treatments for humans or animals are available to date (Tipih and Burt, 2020). With a case fatality rate ranging from 10 to 40%, and only supportive care to control the disease symptoms in patients, CCHF is _with COVID-19, Ebola virus disease and Marburg virus disease, Lassa fever, Middle East respiratory syndrome (MERS) and Severe Acute Respiratory Syndrome (SARS), Nipah and henipaviral diseases, Rift Valley fever, Zika and Disease X_ on the blueprint list of priority diseases on which most research and development efforts should be focused (Freitas et al., 2022; WHO, 2022a).

The epidemiology of CCHF has long been underestimated in Africa. Though not many efforts to understand CCHF have been made on the continent, reports show the evident activity of the virus in animals and ticks. Since the year 2000, at least nineteen African countries have described CCHF outbreaks in humans, showing the growing impact of this disease (Temur et al., 2021; WHO, 2022b). Indeed, Mali, Mauritania, Namibia, Nigeria, Senegal, South Africa, Sudan, and Uganda have each reported at least three human outbreaks from 2010 to 2021 (Zivcec et al., 2017; Boushab et al., 2020; Temur et al., 2021). Very little information is available on CCHF in Central Africa besides small-scale seroprevalence studies in cattle or humans (Guilherme et al., 1996; Sas et al., 2017; Sadeuh-Mba et al., 2018; González Gordon and Bessell, 2022). Grard and colleagues have however described in 2008 a human CCHF case in the Democratic Republic of the Congo (DRC) with the occurrence of CCHFV genotype II (Grard et al., 2011). The aim of this study was to gain greater insight into CCHFV activity by screening domestic ruminants and their attached ticks for the detection of either specific antibodies or the virus in two markets of Yaoundé in Cameroon.

## Materials and methods

### Ethical consideration and authorization

The study protocol was implemented with approval from the Regional Delegation of the Ministry of Livestock, Fisheries, and Animal Industries (MINEPIA), authorizations N°000151/L/MINEPIA/SG/DREPIA-CE/SRDPIA and 00034/L/MINEPIA/SG/DREPIA-CE. Oral consent for blood and ticks sampling was obtained from the animal’s owners.

### Description of the study sites

The study was carried out in two main livestock markets of Yaoundé, Cameroon namely Etoudi (3°55’N, 11°31’36” E) for cattle, and Tsinga market (3°53’55” N, 11°29’30” E) for goats and sheep. The cattle found in Etoudi market arrive from the Adamawa, North, and Far-North regions of Cameroon as well as neighboring countries, to a lesser extent. The small ruminants in Etoudi market come from the northern and western regions of the country. These study sites have been previously described (Sado and Tchetgna, 2022).

### Sample collection and processing

After obtaining consent from the herd’s owners, a questionnaire was administered, and we randomly sampled 10% of the herd. The age of animals was determined using the characteristics of their horns and dentition. Blood and ticks were sampled in June and August 2019, then February and March 2020, and finally March and April 2021. Plasma was obtained after centrifugation at 2500 rpm for 10 min and stored at -20°C until analysis. Ticks were removed manually or with forceps and kept in individual 15mL falcon tubes per animal. Once in the laboratory, the ticks were washed in ethanol 70%v/v, rinsed twice with sterile water, and finally washed in cell culture medium (Minimum Essential Medium, Gibco, Thermo Fisher Scientific, Gloucester, UK). They were subsequently identified using a stereomicroscope (LEICA EZ4E, LEICA Microsystems, Wetzlar, Germany) based on published morphological taxonomic keys (Walker et al., 2003), preserved in RNAlater™ Stabilization Solution (Invitrogen™, Life Technologies, Carlsbad, California, USA) and stored at -80°C until further analysis. To support morphological identification, the tick species was confirmed by molecular analysis of the Cytochrome c Oxidase subunit 1 (*Cox1*) and *16S* rDNA (Lv. et al., 2014; Sado Yousseu et al., 2022).

### Screening of antibodies against CCHFV in the plasma of domestic ruminants

A double antigen Enzyme-Linked Immunosorbent Assay (da-ELISA) was performed to detect specific antibodies directed against the CCHFV nucleoprotein (CCHFV-NP) following the manufacturer’s instructions (Innovative Diagnostics^®^, Grabels, France) (Sas et al., 2018). The test was conducted in 96-well plates that were pre-coated with recombinant purified CCHFV-NP antigens. The anti-CCHFV-NP antibodies if present in the plasma, formed an antigen-antibody complex which will be recognised by a recombinant CCHFV NP antigen-HRP (horseradish peroxidase). Then the absorbance was measured at 450nm using a Biochrom EZ Read 400 ELISA Microplate Reader (ThermoScientific™, Cambridgeshire, Cambridge, United Kingdom). The test was validated when the mean value of the positive control O.D. (OD_PC_) was greater than 0.350 (OD_PC_ > 0.350) and when the ratio of the mean values of the positive and negative controls was greater than 3 (OD_PC_/OD_NC_ >3). Hence, the positivity percentage was computed over the net OD of the positive control.

### Seroneutralization test using the transcription and entry competent virus-like particle system harboring CCHF glycoproteins

We used a reverse genetic approach to produce nanoluciferase (nanoluc)-expressing CCHF virus-like particles, namely transcriptionally and entry competent virus-like particles (tecVLP) (Freitas et al., 2020). Briefly, Huh7.5 cells were seeded in 10 cm dishes and transfected with 3.6µg of pCAGGS-V5-L WT, 1.2µg of pCAGGS-N, 1.2µg of pT7-nLuc, 3µg of pCAGGS-GP, 3µg of pCAGGS-T7, using GeneJammer transfection reagent (Agilent technologies, Santa Clara, California, USA). The transfection media was replaced 6h post transfection. Cells supernatants were harvested 72h post transfection, filtered through a 0.45 μm filter and nanoluc-tecVLP were aliquoted and stored at 80°C before use. Plasmids used for nanoLuc-tecVLP production were described previously (Bergeron et al., 2010; Devignot et al., 2015). For neutralization assays, nanoLuc-tecVLP were incubated with a 100-fold dilution of sera or control antibodies for 1h at 37°C before infection of Huh7.5 cells. Then, 24h post-infection, cells were lysed and nanoluciferase activity was quantified as relative light unit (RLU) using the Nano-glo Luciferase Assay System (Promega, France) following supplier’s recommendations. Cattle sera from France were used as negative controls, and an anti-Gc neutralizing antibody and a serum from an experimentally infected bovine as the positive control. Cut-off value was calculated as mean of negative controls ±2 standard deviation and set at 1.2x10^5^ RLU. To identify nonspecific neutralization, we also incubated each serum with nanoLuc-VSV-G (glycoprotein of the vesicular stomatitis virus (VSV)) pseudotype lentivirus particles and follow the same procedure as described for tecVLP.

### Detection of the CCHFV S segment in plasma and orthonairovirus L segment in ticks

Viral RNA was extracted from 10% of da-ELISA positive plasma samples randomly selected using QIAamp Viral RNA Mini Kit (Qiagen, Hilden, Germany) according to the manufacturer’s instructions. Then, the cDNA was synthesized with a High-capacity cDNA reverse transcription kit (Applied Biosystems, Foster City, California, USA). Real-time RT-PCR targeting a portion of the nucleoprotein within the S segment was performed with TaqMan™ Universal PCR Master Mix kit (Applied Biosystems, Foster City, California, USA) and 400 nM of forward (5’CAAGGGGTACCAAGAAAATGAAGAAGGC3’, position 1068 to 1095) and reverse (5’GCCACAGGGATTGTTCCAAAGCAGAC3’, position 1223 to 1248) primers and 200 nM probe (5’FAM-ATCTACATGCACCCTGCTGTGTTGACA-TAMRA3’) (Wölfel et al., 2007). PCR amplification was completed using a Stratagene Mx3005P qPCR machine (Agilent Technologies, Santa Clara, California, USA).

Additionally, RNA was extracted from pools of three to eight ticks per species and animal using the TRIzol™ Reagent according to the manufacturer (Invitrogen™, Waltham, Massachusetts, USA). Then cDNA was synthesized as described above and RT-PCR was performed using the KAPA Taq PCR Kit (Kapa Biosystems, Wilmington, Massachusetts, USA) and primers described elsewhere targeting the L segment (Forward 5’ ATGATTGCIAAYAGIAAYTTYAA 3’; reverse 5’ ACAGCARTGIATIGGICCCCAYTT 3’) (Honig et al., 2004). The reaction was subjected to a denaturation cycle at 95°C for 5 min followed by 45 amplification cycles at 94°C for 30 s, 56°C for 1min, 72°C for 1min, and a final extension at 72°C for 10 min. PCR amplicons were visualized on a 2% agarose gel containing SYBR™ Safe DNA Gel Stain (Invitrogen™, Massachusetts, USA) and a 100 bp Hyper Ladder™ (Bioline, Thomas Scientific, New Jersey, USA) for an expected size of 445bp. Then, the amplicons were purified using the ExoSAP-IT™ (Applied Biosystems™, Foster City, California, USA) and sequenced at the Microsynth laboratory using the Sanger BigDye terminator technology (Microsynth AG, Germany).

### Phylogenetic analysis of a fragment of the L segment of orthonairoviruses

Orthonairovirus sequences obtained were identified by comparison with different organisms using BLASTN (https://blast.ncbi.nlm.nih.gov/Blast.cgi). Dugbe and Crimean-Congo hemorrhagic fever viruses were identified using a 445 bp fragment of the L segment and deposited in Genbank under the accession number OP292216 and ON564456. A multiple sequence alignment was performed using CCHFV sequence and reference genomes available in GenBank using MAFFT v7 in Unipro Ugene v34.0 (Okonechnikov et al., 2012; Nakamura et al., 2018) and manually edited. The phylogenetic tree was inferred using the maximum-likelihood (ML) method implemented in IQ-Tree v2.2.0. under the TIM2+F+I substitution model obtained with ModelFinder according to the Bayesian Information Criterion (BIC) (Nguyen et al., 2014; Kalyaanamoorthy et al., 2017). The node supports were estimated from 1000 bootstrap replicates. The final tree was read and annotated using FigTree v1.4.4.

### Data analysis

The serological data were analyzed using R software version 4.0.3 for windows *via* RStudio Version 1.3.1093 (RStudio, 2020). Overall seroprevalence with 95% confidence intervals was calculated. Seroprevalence rates were compared between the animal’s origins using the Fisher exact test. The prevalence of infection in ticks was determined and was compared per tick species. Statistical significance was considered at *p*-value < 0.05.

## Results

### CCHFV antibody seroprevalence in the study population

Overall, 756 adult animals (692 males and 64 females) were included in this study, comprising 441 cattle (438 males, 3 females), 168 goats (119 males, 49 females), and 147 sheep (135 males, 12 females). Most cattle were from Cameroon (324/441; 73.47%) but some arrived from Chad (98/441; 22.22%), and Sudan (19/441; 4.31%). In Cameroon, cattle were recorded at the North (43.83%, 142/324), Adamawa (38.58%, 125/324), and Far-North 17.59%, 57/324) regions. All sheep and goats were from the North region of the country (**Table 1**).

**Table 1:** Description of the animal population included in the study.

Country	Region	Cattle		Goats		Sheep		Frequency (%)
		F	M	F	M	F	M	
Cameroon	Adamawa	0	125	0	0	0	0	125 (16.53)
	North	2	140	49	119	12	135	457 (60.45)
	Far-North	0	57	0	0	0	0	57 (7.54)
Chad		0	98	0	0	0	0	98 (12.97)
Sudan		1	18	0	0	0	0	19 (2.51)
Total		3	438	49	119	12	135	756

F, female; M, male.

**Figure 1:**
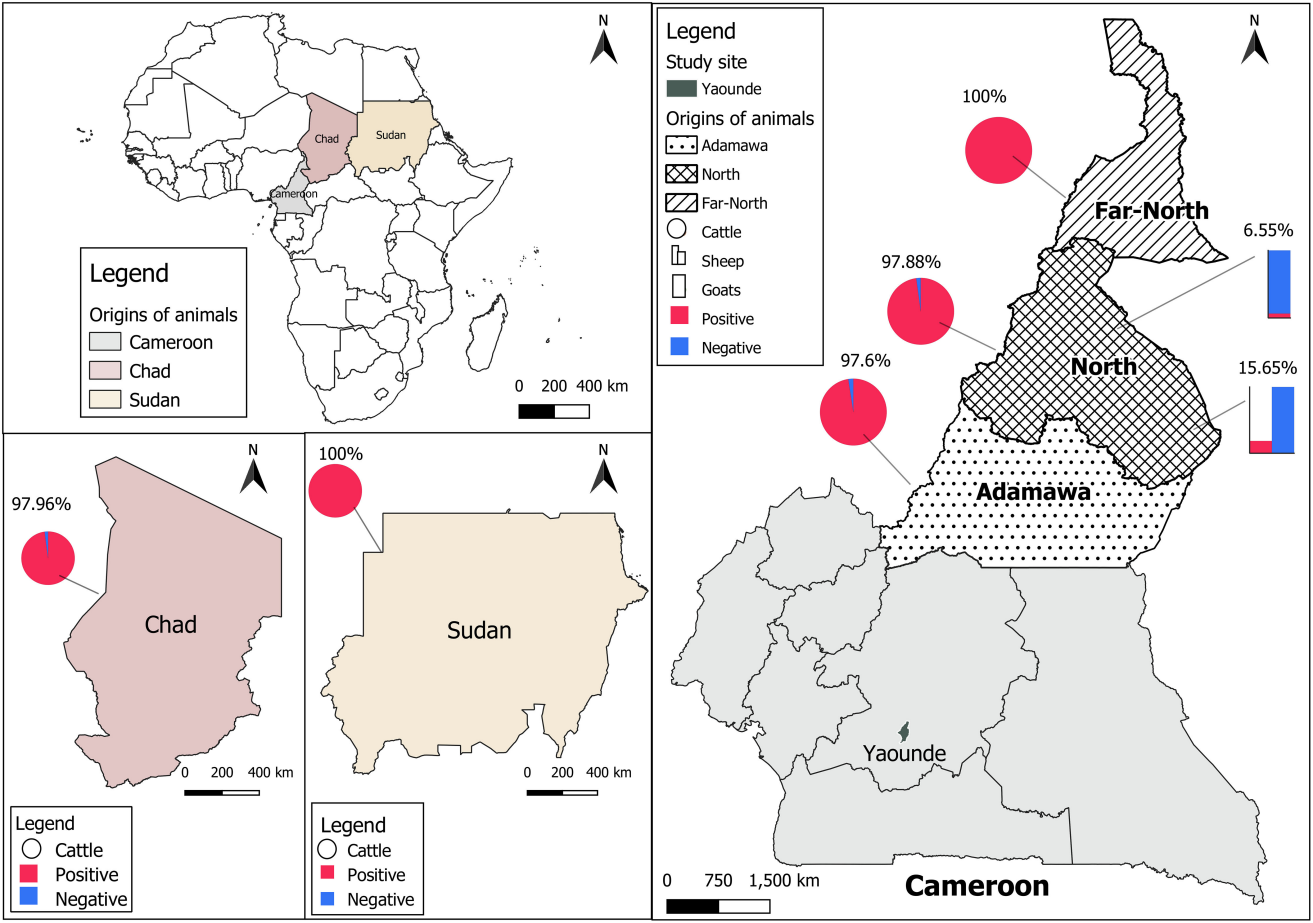
Distribution of CCHFV seroprevalence. Cattle data are presented as pie charts, sheep as histogram, and goats by stacked bars. The positive samples are presented in red and the negative ones are in blue. The prevalence of anti-CCHFV antibodies is presented on each country map.

The CCHFV seroprevalence observed in all animals was 61.77% (467/756, 95% CI: [58.20-65.25]) with the highest seroprevalence in cattle, 98.18% (433/441, 95% CI: [96.46-99.21]), while low seroprevalences were reported in sheep, 15.65% (23/147, 95% CI: [10.18-22.55]) and goats, 6.55% (11/168, 95% CI: [3.31-11.41]). The difference in seroprevalence rate was statistically higher in cattle than in small ruminants (*p*-value < 0.0001, *X^2^* = 594.3; (**Figure 1**). We also observed a slightly higher seroprevalence rate in cattle coming from Sudan (100%, 19/19) compared to Cameroon (98.15%, 318/324) and Chad (97.96%, 96/98), but the difference was not significant (*p*-value < 0.2408, *X^2^* = 2.85). In Cameroon, the highest seroprevalence was found in cattle from the Far-North (100%, 57/57) followed by the North (97.88%, 139/142), and the Adamawa (97.60%, 122/125) but the differences were not statistically significant (*p*-value < 0.2164, *X^2^* = 3.06). No amplification of the S segment was observed by real time RT-PCR on da-ELISA positive samples.

### Seroneutralization of CCHF da-ELISA positive and negative samples

**Figure 2:**
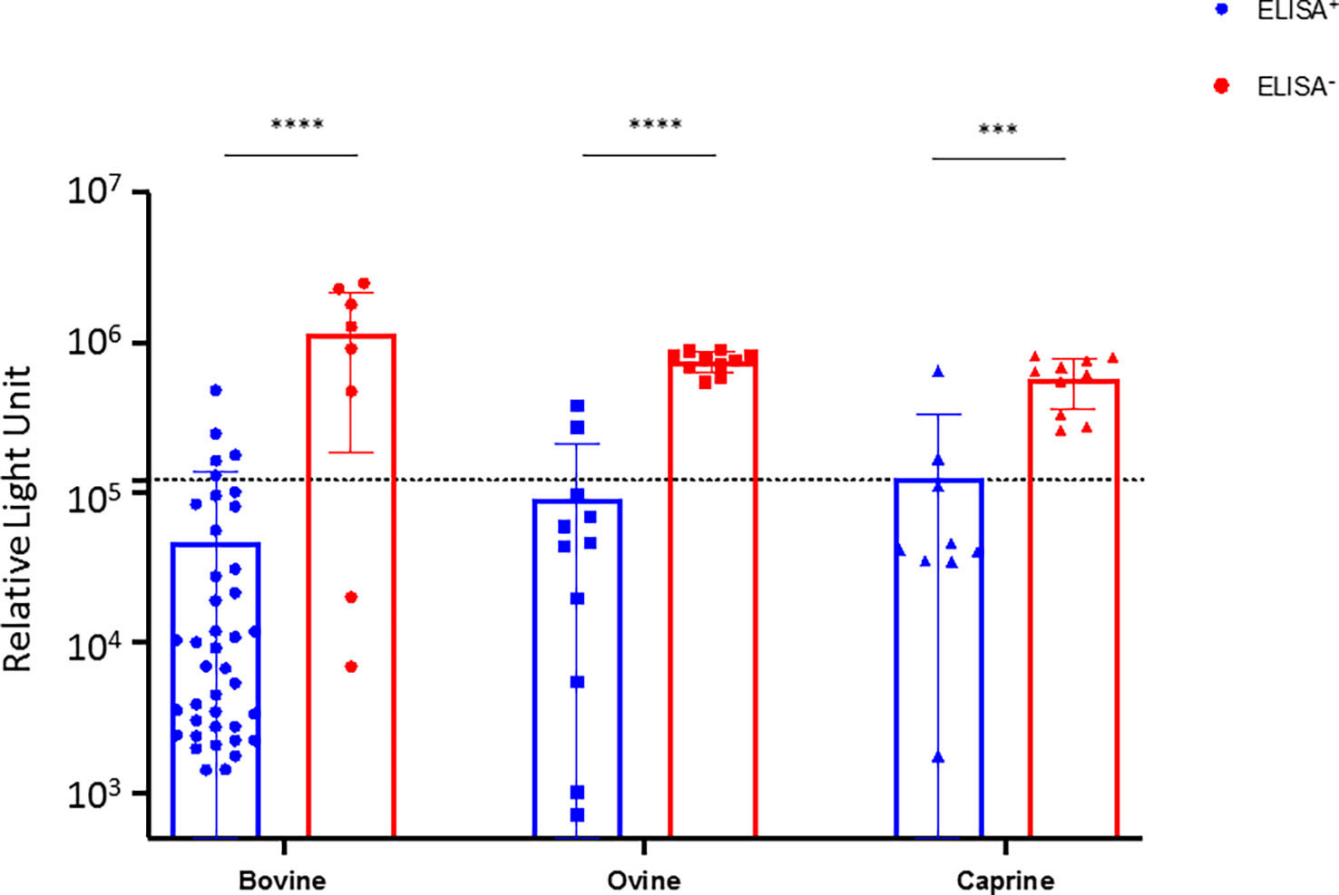
Seuroneutralization assay using the tecVLP system on selected da-ELISA samples. Bovine, ovine, and caprine sera from the ELISA+ (blue) and ELISA- (red) groups were analyzed using a CCHFV tecVLP-based neutralization assay. Neutralizing activities are expressed as RLU (relative light unit). Each dot represents one serum. Dotted line represents the threshold value. Results were statistically analysed using Graph Pad Prism, unpaired t-test. Asterisks show the significant difference. p-value < 0.005.

To confirm the specificity of the results obtained with the da-ELISA test, we performed seroneutralization assays using a CCHFV tec-VLP system. Hence, 40 cattle, 11 sheep, and 9 goats da-ELISA positive samples (da-ELISA+), and 8 cattle, 10 sheep, and 10 goats da-ELISA negative samples (da-ELISA–) were assessed for their seroneutralizing activity. Additionally, 27 bovines sampled in France (CCHFV non-exposed, negative controls) and one experimentally infected bovine (kindly provided by Dr. Loïc Comtet, IDVet, Montpellier) were included in the seroneutralization test. The da-ELISA+ group gave significantly higher neutralization compared to ELISA– of bovine, ovine, and caprine, respectively (mean RLU = 4.62E4 vs 1.2E6; 9.0E4 vs 7.5E5; 1.3E5 vs 5.7E5) (**Figure 2**). All samples from non-exposed bovine (collected in France) were found to be non-neutralizing (mean RLU=9.9E5), in contrast to a serum collected on an experimentally infected bovine (RLU=2.3E3) and an anti-CCHFV-Gc neutralizing antibody (mean RLU=5.7E3, **Supplementary Figure 1**). Additionally, we tested the specificity of the seroneutralizing activity of the serum with a VSV-G pseudotype expressing NanoLuc system. None of the sera exhibited seroneutralizing activity, contrary to an anti-VSV-G neutralizing antibody, further confirming the specificity of the CCHFV tecVLP system (**Supplementary Figure 2**). Overall, we were able to confirm 35 (87.5%), 8 (80%) and 8 (80%) of the positive sera detected with the da-ELISA in bovine, ovine and caprine respectively. Interestingly, 2 bovine sera from the da-ELISA- group showed high seroneutralization (RLU 6.9E3 and 2.0E4, **Figure 2**) which could suggest that some positive individuals may not have been detected by da-ELISA.

### Orthonairoviruses prevalence in ticks screened

Overall, 1500 ticks from cattle, sheep, and goat comprising 3 genera and 11 species were grouped in 300 pools and tested by RT-PCR. The ticks included *Amblyomma variegatum* (22.73%; 61 pools), *Rhipicephalus decoloratus* (19.67%;57pools), *R. microplus* (17.13%; 50 pools), *R. annulatus* (8.47%; 27 pools), *R. sanguineus* (6.27%; 13 pools), *Hyalomma truncatum* (15.13; 48 pools)*, H. rufipes* (5%; 19 pools), *H. nitidum* (2.73%; 9 pools), *H. impetaltum* (2.33%; 12 pools), *H. detritum* (0.27%; 1 pool), and *H. dromedarii* (0.27%; 3 pools). Orthonairovirsues were obtained in one out of 92 *Hyalomma* pools tested (1/92, 1.08%) and one pool of *Amblyomma variegatum* on 61 (1/61, 1.64%). CCHFV was detected in *Hyalomma truncatum* (2.08%, 1/48) and Dugbe virus in *A. variegatum* (**Table 2**). CCHFV and Dugbe virus were detected in ticks sampled from cattle from the Adamawa and North regions of Cameroon in 2019, and 2021 respectively.

**Table 2:** Checklist of the tick species screened for orthonairoviruses and the infection rates detected.

Tick species	Total	Female	Male	Pools	Positive pools (%)	Virus	Genbank accession number
*Hyalomma truncatum*	227	49	178	48	1 (2.08)	CCHFV	**ON564456**
*Hyalomma impeltatum*	35	20	15	12	0	na	na
*Hyalomma rufipes*	75	15	60	19	0	na	na
*Hyalomma dromedarii*	4	2	2	3	0	na	na
*Hyalomma nitidum*	41	9	32	9	0	na	na
*Hyalomma detritum*	4	4	0	1	0	na	na
*Amblyomma variegatum*	341	142	199	61	1 (1.64%)	DUGV	**OP292216**
*Rhipicephalus decoloratus*	295	288	7	57	0	na	na
*Rhipicephalus microplus*	257	255	2	50	0	na	na
*Rhipicephalus annulatus*	127	127	0	27	0	na	na
*Rhipicephalus sanguineus*	94	67	27	13	0	na	na
Total	1500	978	522	300	2 (0.67%)	na	na

na, not applicable; CCHFV, Crimean-Congo hemorrhagic fever virus; DUGV, Dugbe virus. The bold values represent the Genbank accession numbers of the sequences generated in this study.

### Phylogenetic analysis of the L segment of CCHFV

**Figure 3:**
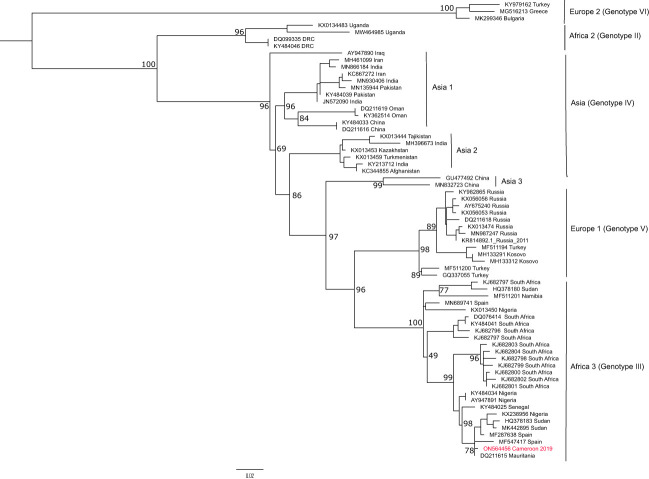
Phylogenetic analysis of the L segment of CCHFV from Cameroon. The maximum likelihood tree was constructedon a 445 bp fragment of the L segment under the TIM2+F+I substitution model and 1000 bootstrap resamplings. Bootstraps are shown only for the main nodes. The CCHFV sequence obtained from Cameroon in this study is highlighted in red. The tree was rooted at midpoint.

The phylogenetic tree constructed with the 445bp fragment of the L segment is concordant with the L segment phylogeny, with a clear distinction of six genotypes grouped per region. The strain from Cameroon clusters within the African III genotype which seems to be widely distributed across sub-Saharan Africa (**Figure 3**). Indeed, the new CCHFV strain forms a distinct clade with strains recently implicated in human CCHFV outbreaks in Sudan, Nigeria, and Spain and seems closely related or identical to a strain isolated in Mauritania in 1984 from *H. rufipes*.

## Discussion

Crimean-Congo haemorrhagic fever is included in the WHO list of top priority diseases in public health emergency contexts on which research and development should be focused (WHO, 2022a). With the same goal, we conducted a cross-sectional survey of CCHFV in livestock and ticks from various locations in Cameroon to evaluate the risk to the local human population. The contact of cattle, goats, and sheep with CCHFV was assessed using a double antigen sandwich ELISA test confirmed by a seroneutralization test using the tecVLP system while ticks were grouped in species-specific pools and screened by RT-PCR.

The CCHFV seroprevalence was high in all tested animals (61.77%) with the highest seroprevalence in cattle (98.18%) as compared to sheep (15.65%) and goats (6.55%). With such alarming and surprisingly high seroprevalences, cross-reaction, as previously described among orthonairoviruses, especially for CCHFV and Hazara virus (Vanhomwegen et al., 2012; Kalkan-Yazıcı et al., 2021) was a possible explanation. Hence, we selected some negative and positive da-ELISA samples for confirmation by seroneutralization, since da-ELISA was not validated for cross reactivity with other orthonairoviruses. However, CCHFV is a biosafety level 4 (BSL-4) pathogen that should be handled in high confinement settings, often not available or affordable by every institution (Weidmann et al., 2016; Organization, 2018). This limitation was overcome using the tecVLP system, an alternative valuable BSL-2 seroneutralization method (Zivcec et al., 2015; Sas et al., 2018). Interestingly, the tecVLP results were concordant with the da-ELISA results, in favour of the high CCHFV seroprevalence observed, even though cross-seroneutralization could occur in case of infection with a closely related virus. Moreover, two sera from the da-ELISA negative group were found seroneutralizing, which could reflect a better sensitivity of the CCHFV tecVLP system. Although da-ELISA and tecVLP seroneutralisation assay results were concordant, logistic and financial constraints preclude the testing of more if not all samples with tecVLP assay.

In Cameroon, the circulation of CCHFV has been documented in humans and cattle but never in small ruminants until now (Sadeuh-Mba et al., 2018; González Gordon and Bessell, 2022). Indeed, González Gordon and colleagues and Sadeuh-Mba and colleagues have described high seroprevalence in pastoral cattle and low seroprevalence in humans in Cameroon respectively (Sadeuh-Mba et al., 2018; González Gordon and Bessell, 2022). The seroprevalence of CCHFV has been shown to be associated with many risk factors including the level of infestation and permanent bites by infected ticks (Adam et al., 2013; Mangombi et al., 2020). Animals may be infested by infected ticks once or recurrently during their lifetime, leading to a permanent activation of their immunity. However, little is known about the longevity of the anti-CCHFV antibody response in non-human vertebrates (Goedhals et al., 2017; Mangombi et al., 2020). This above-mentioned hypothesis is supported by the fact that we have previously reported high tick infestation rates in the cattle compared to small ruminants and a predominance of *Hyalomma* spp. ticks in cattle than small ruminants (Sado Yousseu et al., 2022). Unfortunately, juvenile cattle were not included in the study since sampling was done in markets. That information would contribute to broaden our understanding of recent virus transmission in the country (Nyakarahuka et al., 2018). Our seroprevalence results are higher than those obtained in the subregion in cattle and goats, even in countries with notified human CCHFV cases (Guilherme et al., 1996; Ibrahim et al., 2015; Sas et al., 2017; Mangombi et al., 2020; Oluwayelu et al., 2020; Lysholm et al., 2022). However, our results and those obtained in similar localities in Mali (Maiga et al., 2017; Balinandi et al., 2021) are comparable, supporting the suggestion that the circulation of CCHFV among ticks and vertebrate hosts varies significantly between different locations. Overall, these results may also signal a relatively higher risk of CCHF in Cameroon since CCHFV is mainly transmitted by tick bites and direct contact with bodily fluids on infected animals. This observation also shows the complexity of CCHFV epidemiology and the difficulties when comparing serological data over long periods and across countries.

CCHFV endemic regions overlap the geographic distribution of their *Hyalomma* vectors in Africa, Europe, and Asia. Indeed, *Hyalomma marginatum* is the most studied and known vector of CCHFV in Europe and Asia where it is responsible of the transovarian and transstadial transmission of the virus, ensuring, therefore, its maintenance in nature (Spengler et al., 2016). In Africa, the virus has been detected on numerous occasions in *H. dromedarii, H. truncatum, H. rufipes* and *H. impeltatum* (Swanepoel et al., 1983; Zivcec et al., 2017; Chitimia-Dobler et al., 2019; Kajihara et al., 2021; Schulz et al., 2021) but also in non *Hyalomma* species (Lule et al., 2022). In our study, CCHFV was found in *H. truncatum*, although at a low infection rate but not in *H. rufipes*, *H. impeltatum*, *H. nitidum, H. detritum*, and *H. dromaderii.* The detection of CCHFV genetic material in *Hyalomma* in Cameroon shows the current activity of the virus in the country. However, the role of these ticks in CCHFV maintenance and transmission is yet to be determined, especially their vector competence since we cannot conclude whether they became infected after feeding on these hosts or they were previously infected before infesting the livestock (genetic material present in the blood meal, or infectious viral particle) (Gargili et al., 2017). This concern can be at least partially addressed by screening unfed larvae, nymph, and adult ticks of this species to determine their role in CCHFV transmission in Cameroon.

In this study, we have determined that the CCHFV strain active in Cameroon belongs to the African III genotype on the L segment. Most of the phylogenetic analyses are currently done with the S or M segments for CCHFV classification into genotypes (Fakoorziba et al., 2015; Umair et al., 2020; Monsalve Arteaga et al., 2021). However, studies have also shown that genotypes obtained with the S segment are usually the same as with the L segment, corresponding to the geographic segregation of the virus (Bente et al., 2013). In Central Africa, a great CCHFV genetic diversity has been described, with the occurrence of African II and III genotypes (Grard et al., 2011). Here, we have analysed only a small portion of the L segment (445bp) representing very little genetic information on the virus. Indeed, CCHFV has a high level of genetic recombination and reassortments that can easily distort the classification into genotypes (Grard et al., 2011; Bente et al., 2013; Chinikar et al., 2015; Guo et al., 2017). Therefore, the most conclusive response to the genetic diversity of CCHFV in Cameroon can only be obtained by studying all the three genomic segments.

Given the high seroprevalence observed in animals and the detection of CCHFV in ticks, efforts should be done to increase the survey of CCHF in Cameroon. Indeed, CCHF should be included in the differential diagnosis of acute haemorrhagic fever among at-risk populations in the country. To the best of our knowledge, no human CCHF case has ever been recorded in Cameroon but the disease is known to suddenly emerge among humans in some locations 30 to 50 years apart (Grard et al., 2011).

## Conclusion

In this study, we report high CCHFV seroprevalence in domestic ruminants and virus detection in *Hyalomma* ticks in Cameroon. Although CCHFV is asymptomatic in non-human vertebrates, the high seroprevalence and virus observed raise a public health concern about the occurrence of CCHF among humans, especially among at-risk occupational groups including abattoir workers, farmers, and veterinarians in Cameroon. These findings highlight the suitability of a One Health surveillance system in ticks, wild, and domestic animals that will guide the survey of the disease in humans and include CCHF in the differential diagnostic of acute fevers among at-risk groups in selected regions. Additionally, we have presented here the first genomic sequence of CCHFV in Cameroon, but longer sequences will be required if a greater insight into the genetic diversity and pathogenicity of the CCHFV strains active in the country, is sought. More remains to be done if we wish to understand the epidemiology of CCHFV in Cameroon.

## Data availability statement

The datasets presented in this study can be found in online repositories. The names of the repository/repositories and accession number(s) can be found in the article/**Supplementary Material**. The data are publicly available. Genbank accession number: ON564456 and OP292216. Other data can be found in the supplementary data.

## Ethics statement

The study protocol was implemented with approval from the Regional Delegation of the Ministry of Livestock, Fisheries, and Animal Industries (MINEPIA), authorizations N°000151/L/MINEPIA/SG/DREPIA-CE/SRDPIA and 00034/L/MINEPIA/SG/DREPIA-CE. Oral consent for blood and ticks sampling was obtained from the animal’s owners.

## Author contributions

Conceptualization and study design: HST, PM, VL, CW. Supervision: BK, VL, F-LC, CW. Sample collection and Laboratory work: HST, FSY, NF. Data analysis: HST, FSY, F-LC. Writing- original draft: HST, FSY, VL. Writing – review & editing: HST, FSY, F-LC, NF, BK, PM, RN, VL, CW. All authors contributed to the article and approved the submitted version.
