# Supplementary material for: Molecular and serological evidence of Crimean-Congo hemorrhagic fever orthonairovirus prevalence in livestock and ticks in Cameroon
Source: Front Cell Infect Microbiol. 2023 Mar 28;13:1132495. doi: 10.3389/fcimb.2023.1132495 (PMC10086150; doi:10.3389/fcimb.2023.1132495)
Supplement: Supplementary file 1 [file Presentation_1.pptx]

## Slide 1
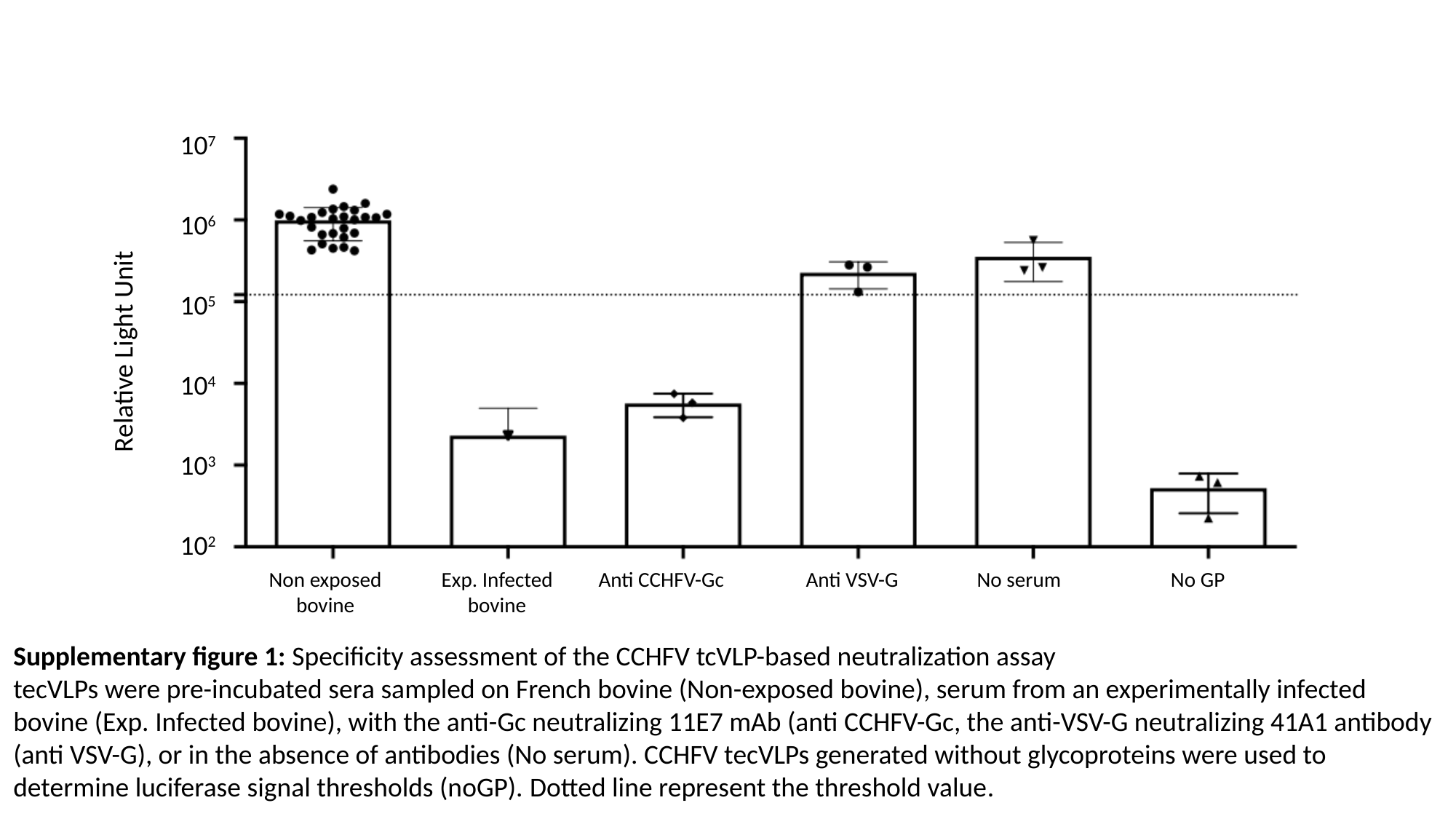

#
107
106
105
Relative Light Unit
104
103
102
Non exposed bovine
Exp. Infected bovine
Anti CCHFV-Gc
Anti VSV-G
No serum
No GP
Supplementary figure 1: Specificity assessment of the CCHFV tcVLP-based neutralization assay
tecVLPs were pre-incubated sera sampled on French bovine (Non-exposed bovine), serum from an experimentally infected bovine (Exp. Infected bovine), with the anti-Gc neutralizing 11E7 mAb (anti CCHFV-Gc, the anti-VSV-G neutralizing 41A1 antibody (anti VSV-G), or in the absence of antibodies (No serum). CCHFV tecVLPs generated without glycoproteins were used to determine luciferase signal thresholds (noGP). Dotted line represent the threshold value.

## Slide 2
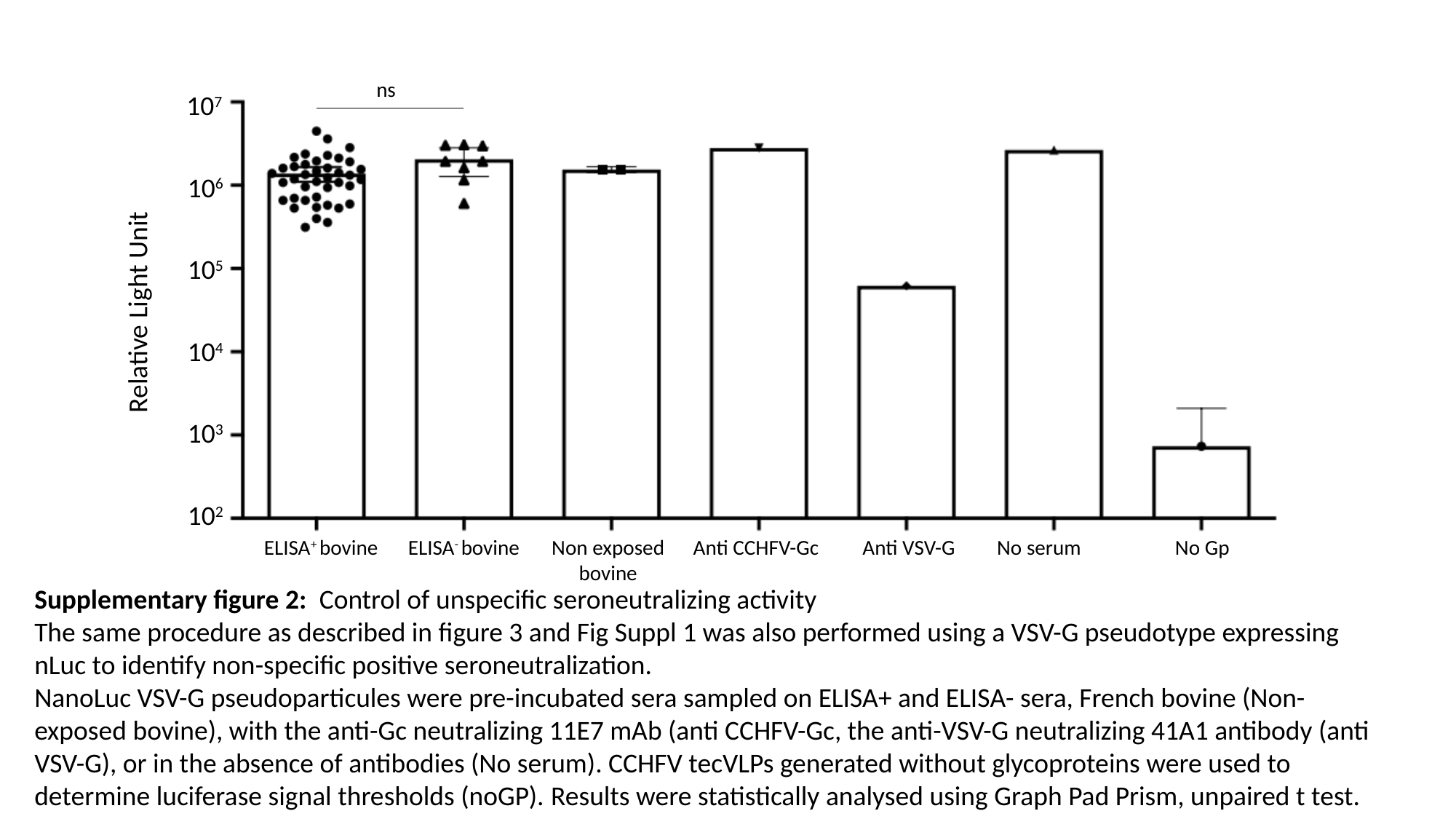

ns
107
106
105
Relative Light Unit
104
103
102
ELISA+ bovine
ELISA- bovine
Non exposed bovine
Anti CCHFV-Gc
Anti VSV-G
No serum
No Gp
Supplementary figure 2: Control of unspecific seroneutralizing activity
The same procedure as described in figure 3 and Fig Suppl 1 was also performed using a VSV-G pseudotype expressing nLuc to identify non-specific positive seroneutralization.
NanoLuc VSV-G pseudoparticules were pre-incubated sera sampled on ELISA+ and ELISA- sera, French bovine (Non-exposed bovine), with the anti-Gc neutralizing 11E7 mAb (anti CCHFV-Gc, the anti-VSV-G neutralizing 41A1 antibody (anti VSV-G), or in the absence of antibodies (No serum). CCHFV tecVLPs generated without glycoproteins were used to determine luciferase signal thresholds (noGP). Results were statistically analysed using Graph Pad Prism, unpaired t test.
